# Finger joint angle and gesture estimation under natural conditions with a soft printed electrode array

**DOI:** 10.1063/5.0270645

**Published:** 2025-10-01

**Authors:** Nitzan Luxembourg, Rufael Fekadu Marew, Dvir Teitelbaum, Dvir Ben-Dov, Hava Siegelmann, Yael Hanein

**Affiliations:** 1 School of Electrical and Computer Engineering, Tel Aviv University, Tel Aviv, Israel; 2 Mohamed bin Zayed University of Artificial Intelligence, Abu Dhabi, United Arab Emirates; 3 Sagol School of Neuroscience, Tel Aviv University, Tel Aviv, Israel; 4 Manning College of Information and Computer Sciences, University of Massachusetts, Amherst, Massachusetts 01003, USA; 5 X-trodes, Herzliya, Israel; 6 Tel Aviv University Center for Nanoscience and Nanotechnology, Tel Aviv University, Tel Aviv, Israel

## Abstract

Innovative methods for finger gesture recognition have been an active research area, with surface electromyography (sEMG) emerging as a promising approach in human-machine interface applications, especially when visual imaging is impractical. However, sEMG-based gesture recognition is highly susceptible to movement artifacts, individual muscle activation, and changes in hand position, making dynamic gesture recognition challenging. While progress has been made in sEMG data collection and analysis, most studies focus on controlled, static hand positions, limiting real-world applicability. This study integrates a soft wearable sEMG sensor, a Video-Vision-Transform model, and motion sensor-based training to predict finger joint angles and recognize gestures across both static and dynamic hand positions. Despite inter-subject variability, results demonstrate differentiation of finger angles and gestures. For the highly performing subject, recognition accuracy reached 0.85 for static and 0.87 for dynamic settings. This work advances sEMG-based gesture recognition, indicating stable performance across tested static and dynamic conditions, suggesting potential suitability for natural and real-world applications.

## I. INTRODUCTION

Automated analysis of finger movements and gestures is a major research topic in human-machine interfaces (HMI).^1,2^ Understanding and accurately interpreting finger movements have significant implications in robotics, virtual reality, prosthetics, and healthcare applications.^3,4^ Finger gesture recognition technologies rely on two primary components: the physical interface (i.e., smart gloves, computer vision, inertial measurement units, and electromyography) and the classification algorithm. The physical interface must offer user convenience, stability, sensitivity, and uninterrupted data acquisition. Recent approaches include smart gloves,^5^ computer vision techniques,^6^ and surface electromyography (sEMG).^7–9^ Notably, sEMG combined with machine learning has emerged as a promising avenue for capturing and interpreting muscle activity associated with finger movements.^8,10,11^

The primary advantage of sEMG is its ability to detect electrical signals generated by muscle contractions without requiring electrodes on the palm or direct hand visualization. Instead, electrodes mounted on the forearm allow unrestricted finger movement. However, several challenges impede the widespread adoption of this technology. These include the sensitivity to mechanical artifacts, cumbersome recording setups, signal dependence on electrode positioning and hand orientation, and overlapping patterns that complicate accurate finger position differentiation.^12^

With the emergence of various deep learning methodologies, extracting meaningful representations from sEMG data has become more robust.^7–10,13–16^ Unlike conventional manually derived features, deep learning models autonomously learn discriminative representations from raw input data, achieving superior performance in complex tasks such as gesture recognition.^17^ Most existing models perform well for one or several fixed-hand positions. However, due to the strong dependence of sEMG data on hand position, models trained only on fixed positions have limited predictive power in real-world applications where the hand and the fingers are expected to move freely. Several recent studies have addressed this challenge and proposed advanced data collection techniques incorporating smart gloves or optical imaging.^12,18^ In particular, Liu *et al.* introduced a novel model integrating three-dimensional (3D) tracking of 21 finger joint angles using sEMG signals.^18^ The Leap Motion sensor was employed as a reference point to evaluate the accuracy and efficacy of the sEMG-based tracking model. Additionally, domain adaptation techniques were utilized to enhance model robustness and mitigate inter-subject variability.^19^

Deep learning has followed a clear trajectory in sEMG data analysis: First, simple convolutional neural networks (CNNs) captured local spatiotemporal motifs but faltered on long-range dependencies.^10,20^ Gated recurrent units such as LSTMs extended the temporal context, yet suffered sequential bottlenecks and gradient issues.^21^ Hybrid CNN-LSTM stacks improved accuracy while suffering from inherent recurrent latency.^22,23^ Transformers resolved this architectural mismatch by applying self-attention over every electrode-time pair in parallel, unifying spatial and temporal cues with high throughput.^24^ Compact Vision-Transformer variants, like CT-HGR, surpassed CNN performance.^11^ Finally, Mahboob *et al.* extended self-attention to continuous 3D kinematic regression.^6^ The video vision transformer (ViViT) can go further by treating the streaming, filtered raw sEMG tensor as a video volume, so it can learn fine-grained finger-angle dynamics without handcrafted features. This ability to exploit the full, noise-reduced spatiotemporal richness of filtered signals makes ViViT a natural choice, which we exploited in our investigation as an architecture for precise, low-latency decoding of finger movements under freely varying hand positions.^25^

Despite advancements in data collection and analysis, relatively little attention has been given to gesture recognition in natural and dynamic conditions. Here, we explore sEMG utility under such conditions. Specifically, subjects performed gestures in random order, with self-selected muscle activation intensity and variable activation onset patterns (sharp to gradual). Unlike previous studies, we permitted hand movement to assess the contribution of mechanical artifacts. Finally, we implemented a decoding model to derive finger joint angles, simplifying training data collection while maintaining validity.

To address limitations in existing technologies, we harnessed a novel sEMG-based system, a ViViT model, and motion sensor-based training. Our study evaluated sEMG-based joint-angle decoder performance under conditions that closely resemble real-world scenarios, including moderate and varied force, randomly ordered gestures, and dynamic hand positions. We demonstrate trajectory-free decoding of continuous hand and finger kinematics using a ViViT architecture and a fully wireless, wearable sEMG system.

Additionally, rest periods were natural and determined by the subjects, allowing them to choose when to rest and resume activities, thereby ensuring ecological validity in our assessments while investigating physiological factors affecting gesture recognition accuracy. The recording system utilizes a conformal electrode array with unprecedented performance in sEMG recording under near-natural conditions. The data analysis approach integrates an analytic algorithm and motion sensor-based training to enhance classification accuracy. The motion-sensor training methodology aims to improve the network's ability to differentiate specific finger positions effectively. We evaluated multiple neural networks to optimize the discrimination of sEMG signals across various gestures. This work advances sEMG-based finger gesture recognition, demonstrating stable performance across tested static and dynamic conditions and suggesting potential suitability for natural movement settings, with prospective applications in prosthetics, virtual reality, and human-machine interfaces.

## II. RESULTS

**FIG. 1.**
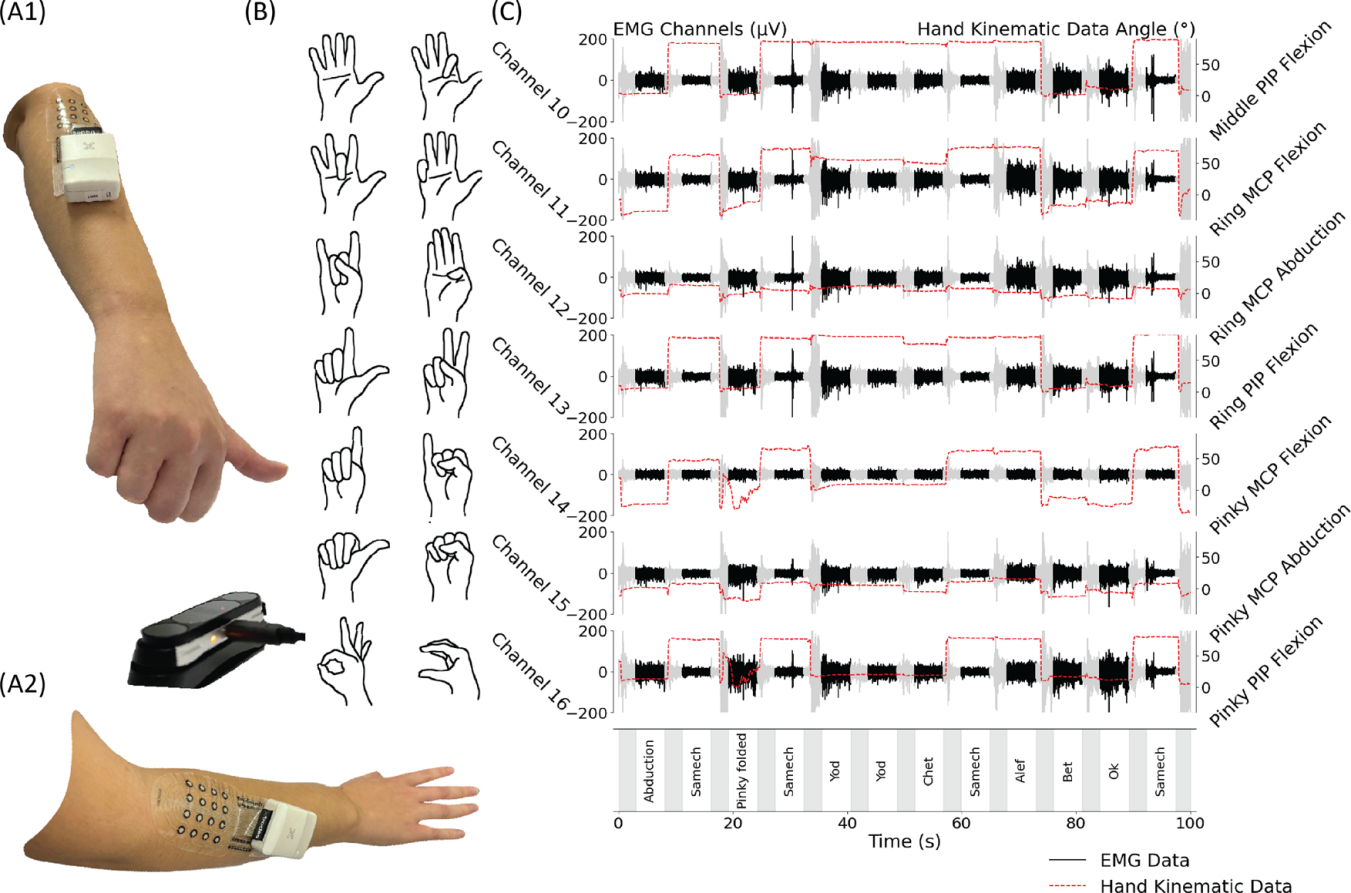
Wireless sEMG and kinematic data acquisition. (a) Experimental setup: A wireless sEMG system with soft printed electrodes placed on the forearm. Motion sensors were utilized to capture hand kinematic angles. (b) The 14 investigated hand gestures, some of which involve overlapping finger positions at varying joint angles. (c) Synchronized sEMG and hand kinematic data. The left y axis represents sEMG amplitude (*μ*V), while the right y axis displays joint angles (°). Recorded gestures are shown in the dark-colored bands, with gray regions indicating posture transition intervals. Hand kinematic data (red dashed lines) are overlaid with sEMG signals (black solid lines).

The experimental setup implemented in this study is illustrated in Figs. 1(a1) and 1(a2). The system consists of a soft electrode array and a compact data acquisition unit (DAU) for real-time sEMG data streaming. sEMG signals were recorded from the arms of healthy subjects. The soft electrode arrays facilitate free hand and body movement while maintaining high signal fidelity. A motion sensor captured finger joint positions to complement the sEMG data. Additionally, visual data were used to train a ViViT model to predict finger joint angles from the sEMG signals.

**FIG. 2.**
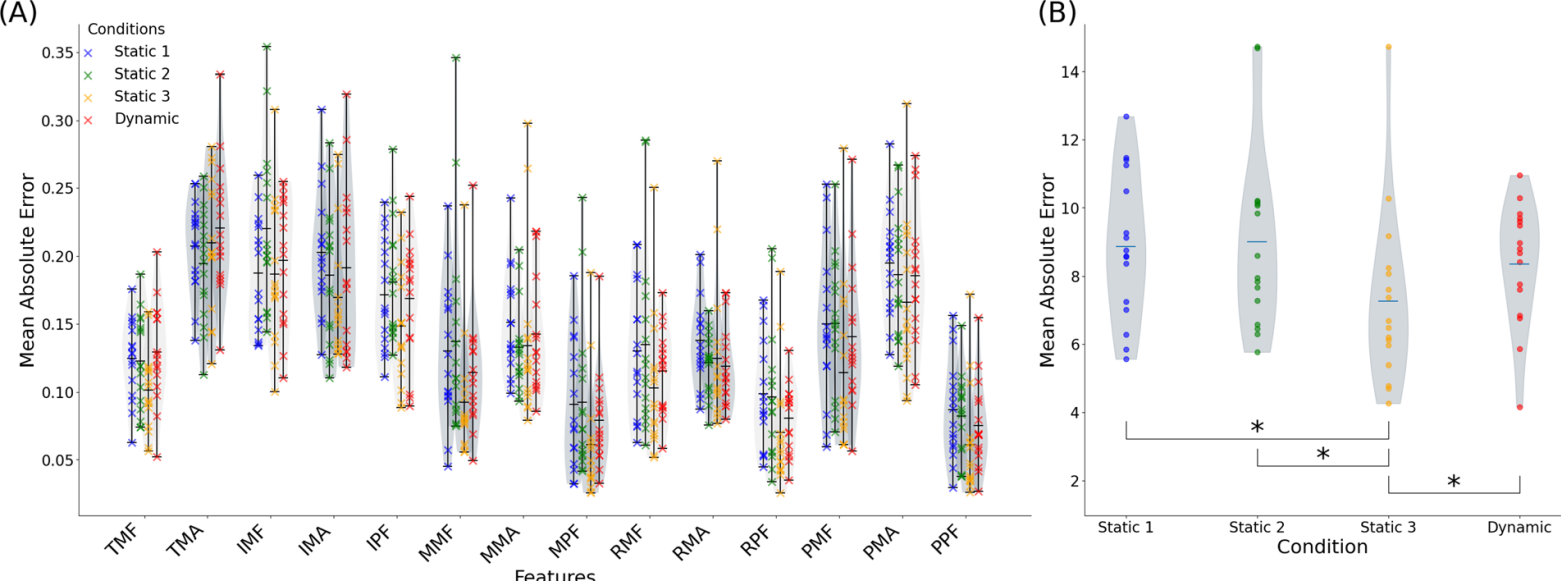
Finger joint angle prediction. (a) Normalized error (in degrees divided by the normal range^26^) between joint angle values from the model's output and motion sensor measurements for the proposed model. (b) Mean absolute error for each setting; asterisk marks significance level using paired t-test p<0.05. Abbreviations: thumb carpometacarpal flexion (TCF); thumb carpometacarpal abduction (TCA); thumb metacarpophalangeal flexion (TMF); thumb metacarpophalangeal abduction (TMA); index metacarpophalangeal flexion (IMF); index metacarpophalangeal abduction (IMA); index proximal interphalangeal flexion (IPF); middle metacarpophalangeal flexion (MMF); middle metacarpophalangeal abduction (MMA); middle proximal interphalangeal flexion (MPF); ring metacarpophalangeal flexion (RMF); ring metacarpophalangeal abduction (RMA); ring proximal interphalangeal flexion (RPF); pinky metacarpophalangeal flexion (PMF); pinky metacarpophalangeal abduction (PMA); pinky proximal interphalangeal flexion (PPF).

sEMG data were collected across three distinct hand positions and conditions (hereafter referred to as *settings*): static 1, hand down (relaxed at the side); static 2, hand straight (arm extended forward, parallel to the ground, and resting on support); static 3, hand up (elbow flexed at 90°, with the palm perpendicular to the ground); and dynamic, subjects freely moved their arms during one of the hand positions while remaining within the sensor's field of view. The dynamic setting was designed to evaluate sEMG signal variations during unconstrained movements, simulating real-world hand gesturing. Bluetooth (BT)-based wireless connectivity ensured high-quality sEMG data acquisition without impeding movement.

Simultaneously with the sEMG data collection, motion sensor data were collected and used as ground truth for training the ViViT model. Each session consisted of 98 events, comprising seven repetitions of 14 distinct finger gestures [Fig. 1(b)]. The first repetition of each gesture was removed, as preliminary analyses indicated that discarding this initial attempt improved performance, likely due to subjects adjusting to the task. Of the remaining repetitions, four per gesture were used for training, one for validation, and one for testing. Gesture presentation was randomized across trials to increase the complexity of the testing paradigm. Importantly, subjects were neither guided to apply strong force nor provided feedback on force application. Typical sEMG and motion sensor data from a single subject are presented in Fig. 1(c). Motion sensor data (in red) show finger joint positions and transitions between gestures. As expected, sEMG activity onset followed motion sensor data, although sEMG amplitude varied even when finger positions remained static. This phenomenon is a reflection of the sEMG data's more informative nature, in particular, its ability to capture force-related differences. Since subjects were not guided to maintain a constant force, sEMG data exhibited greater variability compared to previous studies.^10^ The dark shading of the sEMG data in Fig. 1(c) highlights regions aligning with relative static gestures, typically occurring 1-2 s after movement onset. Occasional brief spikes that appear simultaneously on all electrodes were identified as mechanical artifacts. Their rarity and balanced distribution across training, validation, and test data prevent any systematic bias.

After model training, the ViViT model was used to transform the input data into a 16-dimensional vector representing the key hand kinematic landmarks. The final model outputs were first validated by comparing predicted finger joint angles to motion sensor measurements. The mean absolute prediction error per joint (normalized by its anatomical range^26^) is presented in Fig. 2(a), while Fig. 2(b) shows error distribution across hand positions.

**FIG. 3.**
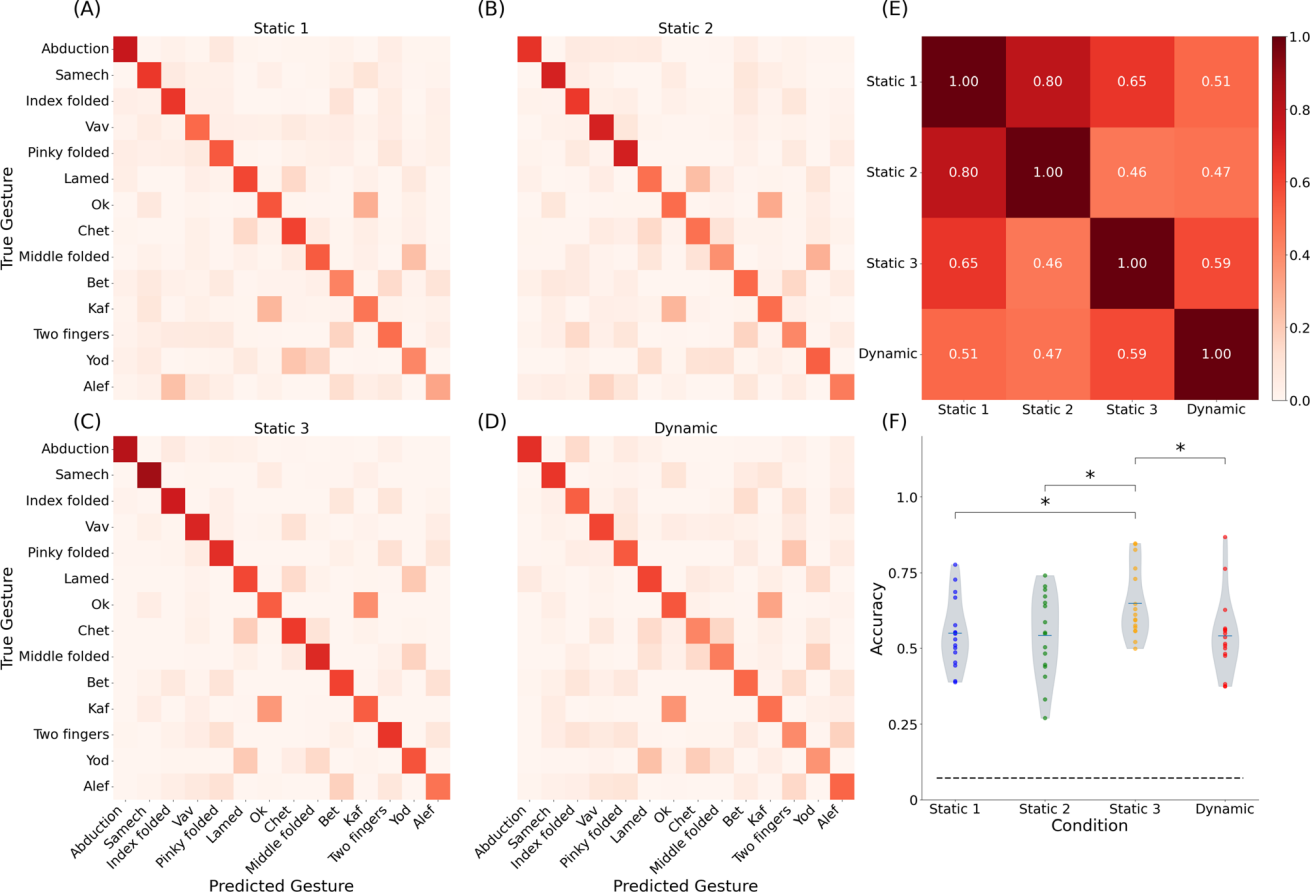
Classification performance of finger gesture recognition across different settings for all subjects (N = 16). Confusion matrices depicting the classification performance for 14 finger gestures under different settings: (a) static 1, (b) static 2, (c) static 3, and (d) dynamic. (e) Per-subject correlation matrix across the four settings, with each cell representing the Pearson correlation coefficient between feature vectors derived from the respective settings. (f) Distribution of average classification accuracy per subject for each setting. The dashed horizontal line denotes the chance level ( $\frac{1}{14}$). Statistical significance is indicated by asterisks ( p<0.05, paired t-test).

To further quantify the model's performance, we examined classification accuracy across the four settings. A classifier was used to map the regression output into a discrete gesture set [14 gestures in Fig. 1(b)]. The confusion matrices for each setting [Figs. 3(a)-3(d)] reveal classification performance across the four tested settings. Figures S2(A)-S2(D) show per-gesture F1 scores, highlighting performance differences across settings. Gestures such as “Abduction” and “Samech” perform consistently well, whereas “Alef,” “Two fingers,” and “Yod” exhibit lower and more variable scores. These trends complement the data presented in Fig. 3. These visible trends may be associated with how different actions are naturally executed (applied force) and the location of electrodes relative to relevant muscles. The per-subject correlation matrix [Fig. 3(e)] was computed from a vector of setting-by-setting accuracy values. While correlations between some settings (e.g., static 1 vs static 2) were relatively high ( r=0.65), others were more modest (e.g., r=0.46), indicating partial rather than complete consistency across conditions. Further substantiation of the correspondence between settings is available in Figs. S2(E)-S2(H). Figure 3(f) further shows the distribution of accuracy per subject, highlighting a significant difference between the static 3 and the other three settings ( p<0.05, paired t-test). A summary of classification accuracy across all settings (after artifact removal) is presented in Table I. Notably, the static 3 setting achieved the highest accuracy (0.648 ± 0.117), while the dynamic setting demonstrated comparable accuracy to static settings, despite increased complexity. Figures S2(E)-S2(H) further reveal that performance drops for gestures like “Alef” and “Two-fingers” in dynamic settings were user-specific, highlighting a crucial limitation.

**TABLE I.** Accuracy across settings and models with equal parameter counts, 14 gestures, and 16 subjects (mean ± SD). Bold values indicate the best performance among the tested models.

Setting	ViViT	CNN + LSTM	CNN	FC
Static 1	0.549±0.114	0.39±0.175	0.306±0.145	0.186±0.1
Static 2	0.542±0.139	0.437±0.157	0.302±0.124	0.226±0.097
Static 3	0.648±0.117	0.478±0.171	0.401±0.117	0.282±0.086
Dynamic	0.540±0.131	0.357±0.174	0.283±0.096	0.218±0.079

**FIG. 4.**
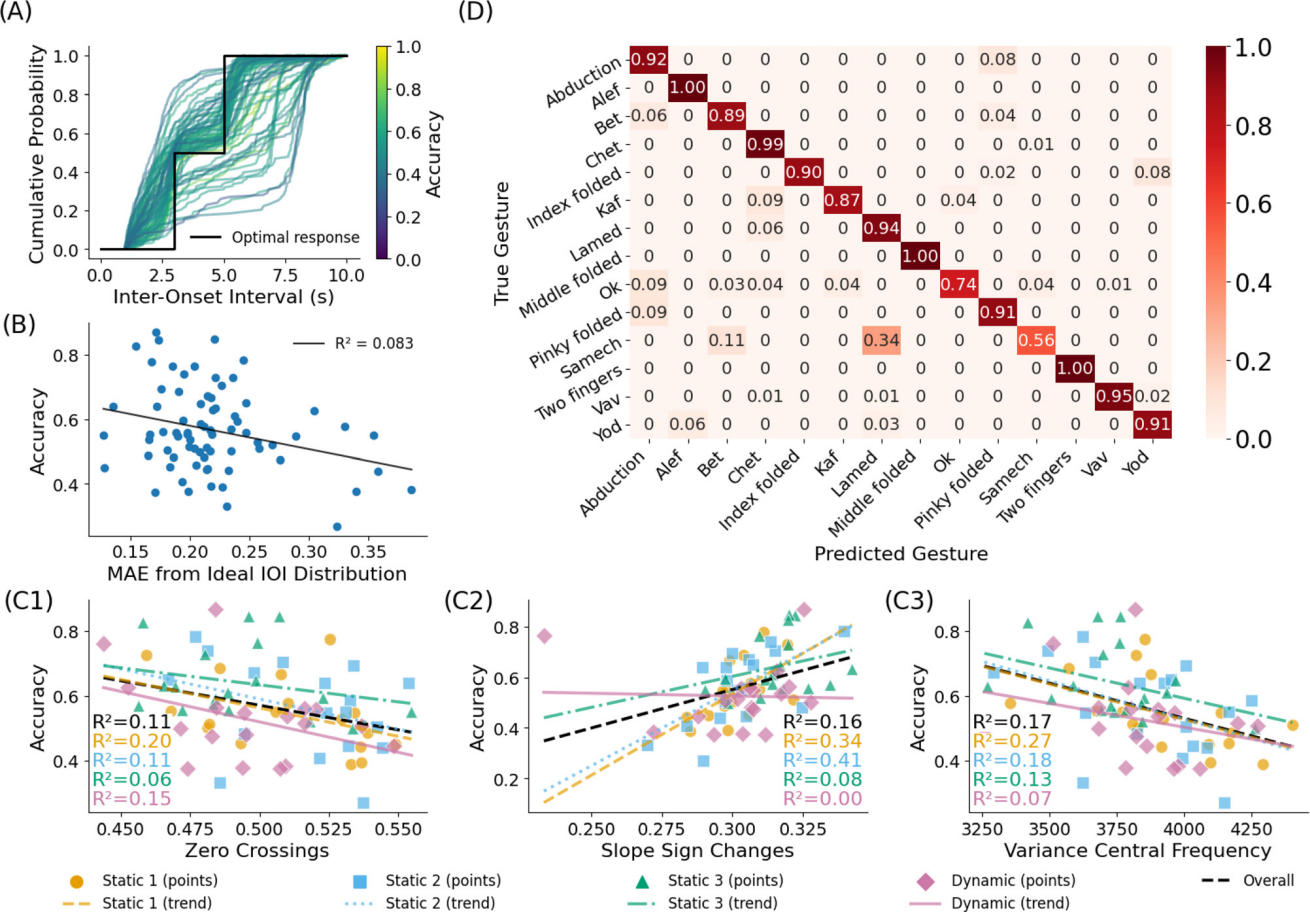
Analysis of gesture recognition accuracy across different signal and behavioral characteristics. (a) Cumulative probability distribution of inter-onset intervals (IOI), with optimal response indicated by the black line. (b) Correlation between accuracy and mean absolute error from the ideal IOI distribution ( $R^{2}=0.083$). Relationship between accuracy and three signal features: (c1) zero crossings, (c2) slope sign changes, and (c3) variance of the central frequency in different settings. (d) Confusion matrix of classification accuracy for all 14 gestures.

The results presented in Fig. 3 show significant variability in model accuracy across subjects, with some subjects reaching accuracy as high as 90% over 14 gestures and others obtaining accuracy as low as 50%. We attribute this variability to the natural conditions in which the data were collected. To illustrate the conditions of the data collection, we plot in Fig. 4(a) the average activation profile for each subject. The activation profile denotes the time when the subject transitioned from one static gesture to another. The black line represents ideal performance with exact and consistent activation timing between each repetition (ideally, each repetition consists of exactly 5 s of gesture activation followed by a 3 s pause, consistently repeated throughout the task). Although subject responses exhibited notable variability and instability, the data in Fig. 4(b) show only a weak link between accuracy and the deviation from the ideal inter-onset interval ( $R^{2}=0.083$).

In Fig. 4(c1)-4(c3), we plot the accuracy vs three statistical measures of the sEMG data: zero crossings, slope sign changes, and variance in central frequency. In fact, the accuracy improves with an increase in slope sign changes but decreases with higher zero crossings and greater variance in central frequency. To examine the impact of gesture execution on accuracy, we conducted a single trial retest with a subject who initially demonstrated moderate accuracy [Fig. 4(d)]. The difference between constrained and unconstrained conditions, and random vs sequential, is readily seen in the raw data (see Fig. S1). Sequential and static conditions are typified by consistent sEMG patterns. Random and dynamic is typified by variability and less consistent sEMG patterns. In the retest, the subject repeated the setting 3 experiment while maintaining 8 s per repetition, with 10 repetitions per gesture. The subject was selected from the initial group and was instructed to apply greater force and to rest between repetitions. As a result, the subject's overall accuracy improved to 89%, representing a 20% increase over the initial session.

## III. DISCUSSION AND CONCLUSIONS

This study focused on studying sEMG-based finger gesture recognition technology toward operation in naturalistic conditions. A major challenge in sEMG data for finger gesture recognition lies in its strong dependency on hand position,^10^ susceptibility to mechanical artifacts, and sEMG signal quality. To address these challenges, we implemented in this investigation several building blocks: (1) a novel wearable and entirely wireless system, (2) naturalistic data collection, (3) a motion sensor as ground truth, (4) multi-position training, and (5) a ViViT-based model to enhance the decoding power. Overall, the strength of this study lies in the diverse gesture set, which includes visually almost overlapping poses. By forcing the model to learn such fine-grained distinctions, we increase its practical relevance compared to previous studies. Our model achieved 0.628±0.097 average accuracy in static settings, comparable to previous reports under the constrained condition depicted in Fig. S1.^15,27–31^

A major challenge in our investigation is susceptibility to mechanical artifacts. Owing to the conformal nature of the electrode and the tight anchoring of the data acquisition unit, the system is relatively immune to these effects, as demonstrated previously in Ref. 32 (Fig. S2).

Major mechanical movements may require the application of mechanical artifact mitigation techniques, such as those employed by Possti *et al.*,^33^ or a comparable approach.

Inter-subject variability remains a challenge, likely driven by differences in muscle activation patterns. Other physiological sources of variability, such as differences in muscle activation patterns, forearm anatomy, or skin impedance, may plausibly affect performance. While machine learning techniques can mitigate some of these variations, it appears that optimizing personalized training remains crucial for high overall performance. In particular, in a preliminary investigation, we observed the effect of applied consistent force as an important parameter, as shown in Fig. 4(d). Prior studies showed that accuracy negatively correlates with body mass index (BMI), probably due to weaker sEMG signals,^7^ consistent with the results discussed here.

Hand orientation also affects muscle activation patterns, with gravity-induced variations further complicating the classification. Figure 3(d) highlights this issue: When the muscles work against gravity, decoding performances are better. Altogether, gesture recognition accuracy is influenced by model type and parameters, number of electrodes, data integrity, and physiological factors. While standard databases (e.g., NinaPro) provide a convenient benchmark, existing databases focus on static settings with relatively strong applied force. In contrast, in this investigation, we explored almost artifact-free data collection in naturalistic conditions.

This study demonstrated the feasibility and potential effectiveness of the proposed approach. Future work should explore physiological sources of variability, such as muscle activation patterns, forearm anatomy, and skin impedance, that may influence performance even when electrode placement is standardized. In addition, efforts should prioritize cross-subject generalization through advanced normalization,^34^ transfer learning,^35^ and zero/few-shot approaches.^36^ Furthermore, expanding dataset size and population diversity will be essential to assess robustness across broader populations, including individuals with neuromuscular conditions. While this work focused on enhancing finger joint angle and gesture recognition accuracy, additional investigations into computational efficiency, real-time processing capabilities, and usability in practical applications are essential.

The results reported here demonstrate good classification accuracy under close to realistic conditions, highlighting the method's robustness across tested conditions. These findings, however, do not yet confirm generalization to unseen conditions or subjects. Future work will explicitly examine cross-condition and cross-subject generalization.

Enhancing subject training helps improve accuracy results to reach high accuracy values. Moreover, the system's reliance on sEMG and relatively simple motion sensors contributes to its cost efficiency, broadening the potential for practical adoption. Altogether, these findings lay the groundwork for more accessible applications, paving the way for further exploration and refinement of sEMG-driven finger gesture recognition in real-world scenarios.

## IV. METHODS

### A. Data acquisition

#### 1. sEMG system

The study employed dry carbon electrode arrays from X-trodes Inc. Each array consisted of 16 electrodes (4.5 mm in diameter) arranged in a 4 × 4 matrix. These arrays were fabricated using screen printing of carbon electrodes and silver conductive traces onto a flexible and thin polyurethane (PU) film, as detailed in Ref. 37. To enhance durability and adhesion, an additional layer of double-sided adhesive PU film is utilized for passivation and secure skin attachment. As the entire electrode array is built on ultra-thin support, the electrodes conform with the skin, allowing stable signal transduction without the need for a gel.^38^

Data were recorded with a miniature wireless data acquisition unit (DAU, X-trodes Inc.), developed to allow electrophysiological measurements under natural conditions. The DAU supports up to 16 unipolar channels with a sampling rate of 4000 S/s, 16-bit resolution, an input range of ±12.5 mV, and an input impedance of $10^{7}\;\Omega$. A 620 mAh battery supports DAU operation for up to 16 hr. A Bluetooth (BT) module is used for continuous data transfer to a personal computer (PC).

#### 2. Leap system

The Leap Motion Controller 2 (Ultraleap Hyperion) was used for hand tracking. This device tracks hands within a 3D interactive zone of up to 110 cm, with a 160° field of view, and can identify 27 distinct hand elements. The controller features high IR sensitivity and operates at a maximum frame rate of 120 fps.^39^ We have extracted the following joint angles: TCF, TCA, TMF, TMA, IMF, IMA, IPF, MMF, MMA, MPF, RMF, RMA, RPF, PMF, PMA, and PPF. Notably, we observed low variability in the TCF and TCA measurements, likely due to limited visibility and depth perception issues inherent in camera-based tracking, suggesting these particular measurements should be interpreted with caution.

#### 3. Data collection

A total of 16 healthy subjects (ages 21-30) participated in the study. The study adhered to the ethical guidelines for research involving human participants and was approved by the Tel Aviv University Ethics Review Board (Approval No. 0004877-3). Informed consent was obtained from all participants in accordance with institutional requirements. Electrode arrays were placed on the participant's dominant hand on the region of the extensor digitorum muscle, identified as the participant flexed the muscle. During the experiment, subjects were shown pictures of the gestures and were requested to perform these gestures at the sound of an auditory cue. The auditory cue sets the timing for the annotation in data (gray vs dark regions in Fig. 1). Most subjects initiated finger movement before the auditory cue.

Participants performed 14 distinct finger gestures, repeated seven times in a random sequence. Each gesture was maintained for 5 s, followed by a 3 s pause. Participants performed the procedure under four different settings. Static 1: hand held down straight with the muscles completely relaxed; static 2: hand extended 90° forward with the palm relaxed; static 3: hand folded upwards with the palm relaxed; and dynamic: one of the previous settings while permitting hand movement within the camera's detection range. Dynamic conditions were approximately 150° horizontally, 120° vertically, and 60 cm away (corresponding with the motion sensor's effective zone), with several seconds for a full range movement. The movements were displayed to the subject in real time on a PC screen, as detailed in Ref. 40. The first repetition of every gesture was removed due to noticeably poorer performance quality, the fifth repetition was designated for testing, the fourth for validation, and the remaining repetitions were used for training. sEMG and Leap Motion data were recorded simultaneously. Python code was used to annotate the start and finish times of each gesture.

Data quality is a major potential issue, in particular for dynamic conditions as those described in this investigation. However, owing to the design of the wearable system used in this study, mechanical artifacts in our data are minimal.^32^ To address concerns regarding data quality, the following measures were applied: Data were visually examined during data collection to validate proper signal and minimize line interference and mechanical artifacts. Second, after data collection, the data were visually examined again. Figure S1 shows typical sEMG data typified by almost no apparent mechanical artifact. Finally, offline data analysis was performed with data segments that did not include transient actions to further minimize concerns regarding mechanical artifacts. Real-time analysis would mandate automatic real-time data evaluation.

### B. Signal processing

#### 1. Filtering

Power-line noise was removed using 50 and 100 Hz comb filters. A fourth-order Butterworth high-pass filter (20 Hz cutoff) was used to eliminate non-sEMG signals using the SciPy Python library.

The hand kinematic model was filtered using a Savitzky-Golay filter, implemented in the SciPy Python library, with a polynomial order of 1 (i.e., weighted moving average) and a window size of 502 samples to eliminate apparent non-biological movements caused by estimator noise. Finally, each sEMG channel was standardized by applying per-channel z-score normalization (mean-centered and scaled to unit variance) before further analysis.

#### 2. Artifact removal

To further refine classification performance, data were preprocessed to remove artifacts and outliers. Specifically, to eliminate outliers resulting from poor performance (i.e., mistakes and corrections), we computed the median hand kinematic angles for each interval and retained only those samples where the maximum deviation of any joint angle from its respective median was less than 15°, a process that filtered out a mean of 0.51% of the data (with a standard deviation of 0.56%) across all subjects and intervals, ultimately leading to improved accuracy.

#### 3. Segmentation

A rolling window approach (512 ms window, 2 ms stride) was applied. This ensures each window captures a consistent segment of the sequence, and relevant features are maintained within each window. Even with substantial overlap between windows, empirical results show that a 2-ms stride converges faster and achieves higher accuracy than larger strides, while early stopping keeps training efficient and prevents overfitting. The resulting input is then transformed into $\hat{x}\in R^{LXSXC}$ where $L=\frac{N-S}{t}$, S is the window size, and t is the step size. Then the channels were placed in a grid in accordance with their position on the sensor, resulting in $\hat{x}\in R^{LXSXWXH}$, where WXH is the width and height of the grid, which in this study equal 4×4.

### C. Video vision transformer

To enhance sEMG-based finger gesture recognition, we employ a factorized self-attention video vision transformer (ViViT) approach,^25^ implementing it using the PyTorch framework, as illustrated in Fig. S3. We utilized the ViViT-pytorch repository^41^ as the foundation for our implementation. By leveraging the electrode array's grid-like configuration, we treat the sEMG measurements as sequences of image-like patches. This perspective allows us to apply visual transformer techniques for capturing both the spatial organization of the electrode layout and the temporal evolution of the signals. The architecture first uses a patch embedding layer to represent the sEMG data, followed by multiple transformer encoder layers to extract complex spatiotemporal features. A final fully connected layer then maps the learned representations into a 16-dimensional vector of finger joint angles, which can then be fed to the downstream classifier.

#### 1. Classification

To comprehensively assess the efficacy of our proposed model, we conducted a downstream classification task. In this evaluation, the model's performance was tested by employing its 16-dimensional joint angle output as input for the extra trees classifier, implemented in the Python scikit-learn library.

#### 2. Augmentations

To improve generalization and mitigate overfitting, we apply a data augmentation procedure that first silences a subset of the channels with a probability of 0.5 for a duration uniformly sampled between 6 and 400 ms. After this silencing step, we added Gaussian noise with a standard deviation of 0.1 to the normalized signal. We also experimented with the augmentation technique proposed by Tsinganos *et al.*^42^ but found that our approach achieved higher performance for the current dataset.

## SUPPLEMENTARY MATERIAL

See the supplementary material for the following: EMG and hand-kinematic signals during unconstrained gestures and during force-plus-rest instructions delivered in random or sequential order (Fig. S1); per-gesture F1 performance and inter-subject variability across acquisition settings (Fig. S2); and architecture of the factorized self-attention video vision transformer (ViViT) (Fig. S3).

## Data Availability

The data that support the findings of this study are available from the corresponding author upon reasonable request.
